# Methodological considerations of electron spin resonance spin trapping techniques for measuring reactive oxygen species generated from metal oxide nanomaterials

**DOI:** 10.1038/srep26347

**Published:** 2016-05-19

**Authors:** Min Sook Jeong, Kyeong-Nam Yu, Hyun Hoon Chung, Soo Jin Park, Ah Young Lee, Mi Ryoung Song, Myung-Haing Cho, Jun Sung Kim

**Affiliations:** 1 R&D Center, Biterials, Goyang-si, Republic of Korea; 2 Department of Chemical & Biomedical Engineering, Cleveland State University, Cleveland, OH, USA; 3 Department of Obstetrics and Gynecology, Seoul National University College of Medicine, Seoul, Republic of Korea; 4 Laboratory of Toxicology, College of Veterinary Medicine, Seoul National University, Seoul, Republic of Korea; 5 Graduate Group of Tumor Biology, Seoul National University, Seoul, Republic of Korea; 6 Advanced Institute of Convergence Technology, Seoul National University, Suwon, Republic of Korea; 7 Graduate School of Convergence Science and Technology, Suwon, Republic of Korea; 8 Institute of GreenBio Science Technology, Seoul National University, Pyeongchang-gun, Republic of Korea

## Abstract

Qualitative and quantitative analyses of reactive oxygen species (ROS) generated on the surfaces of nanomaterials are important for understanding their toxicity and toxic mechanisms, which are in turn beneficial for manufacturing more biocompatible nanomaterials in many industrial fields. Electron spin resonance (ESR) is a useful tool for detecting ROS formation. However, using this technique without first considering the physicochemical properties of nanomaterials and proper conditions of the spin trapping agent (such as incubation time) may lead to misinterpretation of the resulting data. In this report, we suggest methodological considerations for ESR as pertains to magnetism, sample preparation and proper incubation time with spin trapping agents. Based on our results, each spin trapping agent should be given the proper incubation time. For nanomaterials having magnetic properties, it is useful to remove these nanomaterials via centrifugation after reacting with spin trapping agents. Sonication for the purpose of sample dispersion and sample light exposure should be controlled during ESR in order to enhance the obtained ROS signal. This report will allow researchers to better design ESR spin trapping applications involving nanomaterials.

To further promote industries that rely on nanotechnology, it is important to understand not only the characteristics and potentials of such materials, but also their toxicity. The field of toxicology has produced a wealth of information relating to the toxicity or toxic mechanisms of nanomaterials, which have expanded and refined the industrial uses of such materials. In recent decades, these efforts have been highly beneficial to understanding the toxicities and toxic mechanisms of various nanomaterials and to safely and responsibly develop their related technologies. Nevertheless, nanomaterials have many different forms and applications, and this diversity must be considered when studying their toxicity. In particular, in the initial and current state of nanotoxicology, various nanomaterial properties such as size, shape, and charge must be considered due to their pivotal roles in influencing material toxicity. Therefore, determining the physicochemical properties of nanomaterials is critical in evaluating their overall toxicity. In the relevant biological and physicochemical analyses, it is essential to not only produce relevant data but to understand the limitations of existing methods.

Reactive oxygen species (ROS) are highly reactive substances that are potentially toxic to living organisms. They can cause detrimental effects in cells through oxidative damage of biomolecules (proteins, lipids, and nucleic acids) or through disruption of cell signaling pathways^1^. ROS is considered one of the most important sources of toxic mechanisms in various nanomaterials. Many reports suggest that the entrance of nanomaterials into the cell may lead to the formation of ROS. In addition, the formation of ROS in the cell can induce toxicity through a number of complicated pathways^2^,^3^,^4^. Therefore, it is important to measure and quantify ROS formation in order to understand ROS-related toxicity in nanomaterials^5^. ROS typically have a short lifespan, making their direct detection and determination very difficult and often impossible^6^,^7^. The use of fluorescent probes, such as dichlorodihydrofluorescein and hydroethidine, as well as dihydrorhodamine and chemiluminescent methods, provides simple ways of detecting free radicals and ROS in cellular systems. Nevertheless, these methods have limitations and many sources of artifacts^8^,^9^,^10^.

Electron spin resonance (ESR) (also called electron paramagnetic resonance or EPR) is a powerful technique for studying chemical species that have one or more unpaired electrons. ESR spectroscopy has become a direct and potent (robust-Rev1) method for detecting free radicals that are chemically generated or formed in biological systems, and in nanotoxicology this technique has been employed for detecting ROS^11^,^12^,^13^. ESR techniques with various spin trapping agents are used to detect specific free radicals and to produce a relatively stable and distinct spin adduct that can be identified and quantified by ESR^11^. Although ESR with spin trapping agents is a very robust and valuable method of detecting ROS, selection of the correct specific type of spin trapping agent, sample preparation method, and incubation time between spin trapping agents and target materials must be addressed in greater detail. He *et al*., for example, contended that light exposure may affect the formation of ROS in metal nanoparticles, and that it also influences the formation of free radicals^10^,^14^. Moreover, ultrasound was reported by Makino, Mossoba and Riesz to affect overall sample preparation^15^. In this study, we suggest methodological considerations to follow in regards to the magnetism of nanomaterials, incubation time of spin trapping agents, and various other factors that can lead to distortion of ESR signals of nanomaterials such as light exposure and sonication, which are often used carelessly in ESR for measuring the ROS generated from metal oxide nanomaterials.

## Results

### Spin adduct stability as a function of incubation time

**Figure 1:**
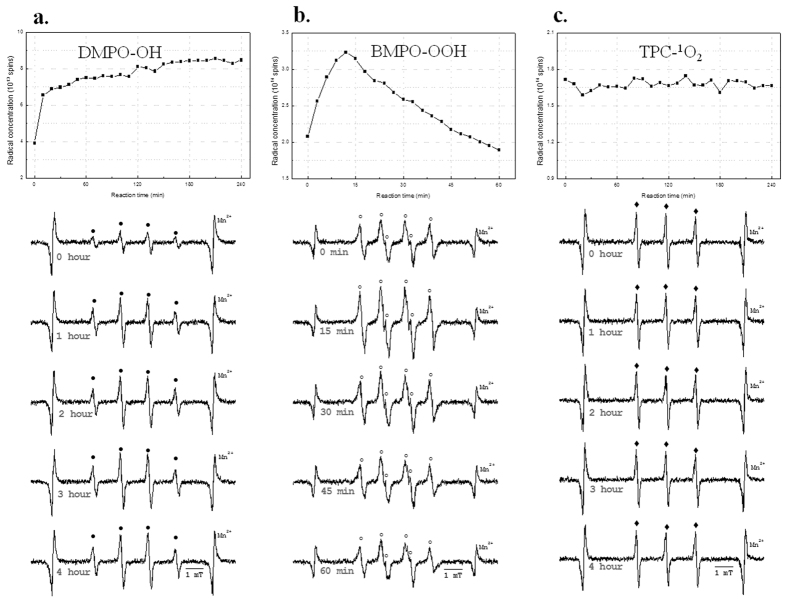
Stability reviews of each spin adduct according to incubation time in each positive control system. (**a**) Stability review of the DMPO-OH adduct generated by the Fenton reaction in the presence of DMPO, (**b**) Stability review of the BMPO-OOH adduct by the hypoxanthine–xanthine oxidase system in the presence of BMPO, (**c**) Stability review of the TPC-^1^O_2_ adduct generated by the Rose Bengal photosensitizer in the presence of TPC.

We analyzed the stability of the spin adduct as a function of the incubation time in a positive control system (Fig. 1). In the case of the DMPO-OH adduct, the signal intensity increases until approximately 150 min in the Fenton reaction system. From 150 min to approximately 4 h, the signal intensity is saturated (Fig. 1a). In the case of the BMPO-OOH adduct, the signal intensity increases until approximately 12 min, and then slowly decreases over time in the hypoxanthine-xanthine oxidase system (Fig. 1b). However, the TPC-^1^O_2_ adduct is very stable until approximately 4 h (Fig. 1c). Based on our results, determination of the incubation time between the spin trapping agent and test materials may be important in future quantitative analysis. These results allow us to determine an optimal reaction time for spin trapping reactions, respectively.

### TEM characterization of test nanomaterials

**Figure 2:**
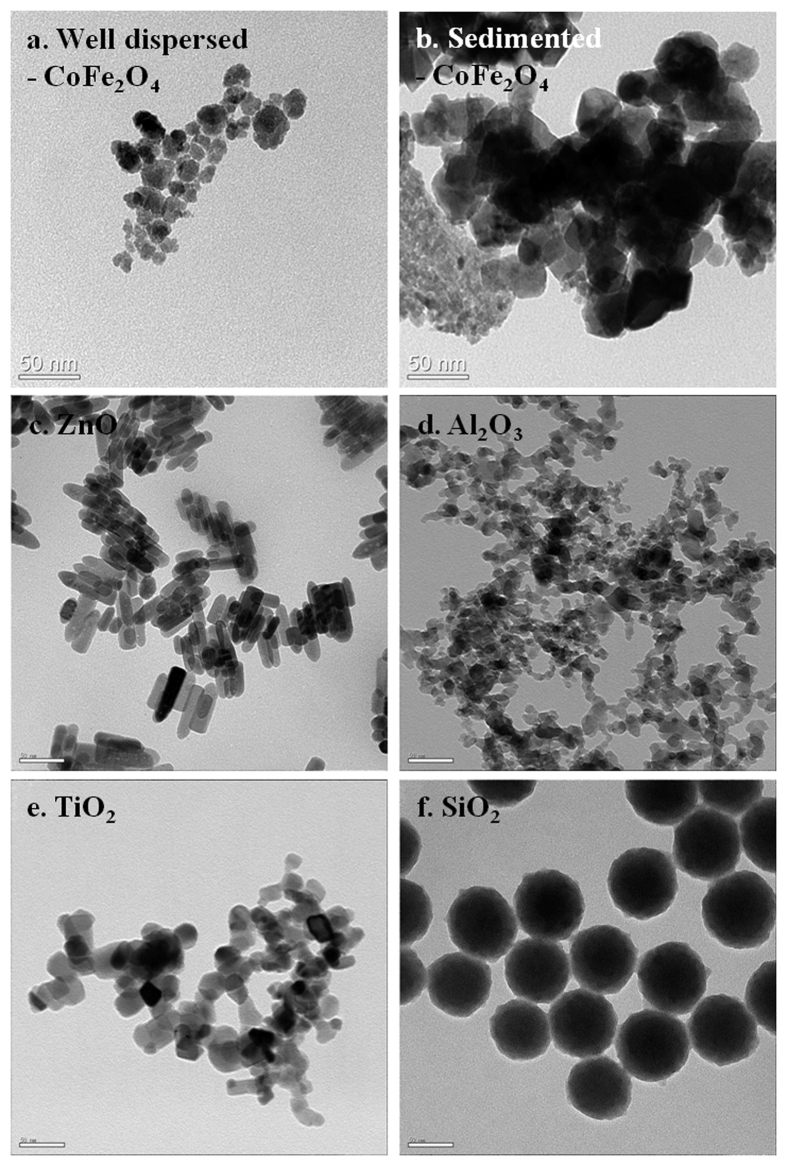
TEM images of metal oxide nanomaterials. (**a**) Well-dispersed CoFe_2_O_4_ (20.4 ± 10.3 nm), (**b**) Sedimented CoFe_2_O_4_ (48.3 ± 9.7 nm), (**c**) ZnO nanorods (width: 16.2 ± 1.3 nm, length: 50.8 ± 11.8 nm), (**d**) Al_2_O_3_ (15.6 ± 5.4 nm), (**e**) TiO_2_ (26.5 ± 6.7 nm), (**f**) SiO_2_ (72.9 ± 4.0 nm).

Transmission electron microscopy (TEM) analysis was performed to confirm the particle morphology and size of the nanomaterials. As shown in Fig. 2, ZnO nanomaterials have a rod-like shape (width: 16.2 ± 1.3 nm, length: 50.8 ± 11.8 nm), SiO_2_ nanomaterials are spherical (72.9 ± 4.0 nm), and the shapes of well dispersed-CoFe_2_O_4_ (20.4 ± 10.3 nm), sedimented-CoFe_2_O_4_ (48.3 ± 9.7 nm), TiO_2_ (26.5 ± 6.7 nm) and Al_2_O_3_ (15.6 ± 5.4 nm) nanomaterials are polygonal.

### Magnetic nanomaterials and centrifugation

**Figure 3:**
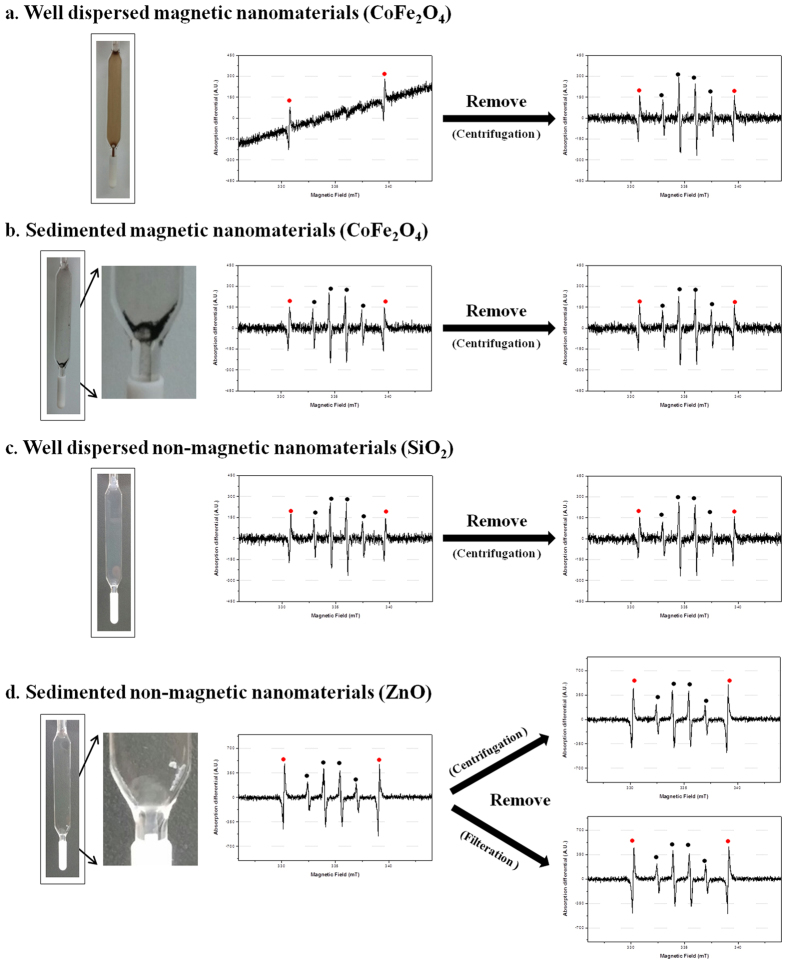
ESR spectra confirming the removal effect of nanomaterials on sample preparation. (**a**) Well-dispersed magnetic nanomaterials (CoFe_2_O_4_), (**b**) Sedimented magnetic nanomaterials (CoFe_2_O_4_). (**c**) Well-dispersed non-magnetic nanomaterials (SiO_2_), (**d**) Sedimented non-magnetic nanomaterials (ZnO). CoFe_2_O_4_ and SiO_2_ nanomaterials were detected in positive control system and ZnO nanomaterials were detected without positive control system. The nanomaterials were removed by centrifugation (at 12700 × g for 10 min) or filtration (with 0.2 μm pore size).

Nanomaterials have many physicochemical properties; among them, magnetism can impact the ESR signal of the material. We compared the ESR signal before and after removing nanomaterials by centrifugation or filtration in the case of CoFe_2_O_4_ (a magnetic nanomaterial) in a positive control system. Because ESR signals of CoFe_2_O_4_ samples were not detected based on hydroxyl radical signals (DMPO-OH adducts) after mixing the nanomaterials and spin trapping agent (DMPO) for 3 h (data not shown), samples were detected using a positive control system. ESR signals were distorted in the case of well-dispersed CoFe_2_O_4_ (Fig. 3a). This ESR signal was tilted to one side because of contributions from both the spin adduct spectrum and a small portion (335 ± 10 mT) of the very broad (few hundred mT) intrinsic ESR spectrum of the unpaired electrons from CoFe_2_O_4_. No ESR signal was present for DMPO-OH adducts (Quartet with 1:2:2:1 signal intensity) despite the positive control system. However, after removing the well-dispersed CoFe_2_O_4_ by centrifugation, the ESR signal was not tilted and DMPO-OH adducts were detected.

In the case of sedimented CoFe_2_O_4_, there is no distortion of the ESR signals (Fig. 3b). Based on this result, we conclude that well-dispersed nanomaterials with magnetic properties should not be directly applied in ESR due to interference issues (Fig. 3a).

To evaluate the interference effects of non-magnetic nanomaterials (ZnO and SiO_2_), ESR spectra were collected before and after removing the nanomaterials by centrifugation or filtration. Here, there is no difference in ESR signal for samples with and without non-magnetic nanomaterials (Fig. 3c,d). Based on our results, there is no adverse effect of removing nanomaterials after incubating the spin trapping agent; therefore we suggest that removal of well-dispersed nanomaterials having magnetic properties by centrifugation could be applied to ESR analysis.

### Effects of light exposure during sample preparation on ESR spectra

**Figure 4:**
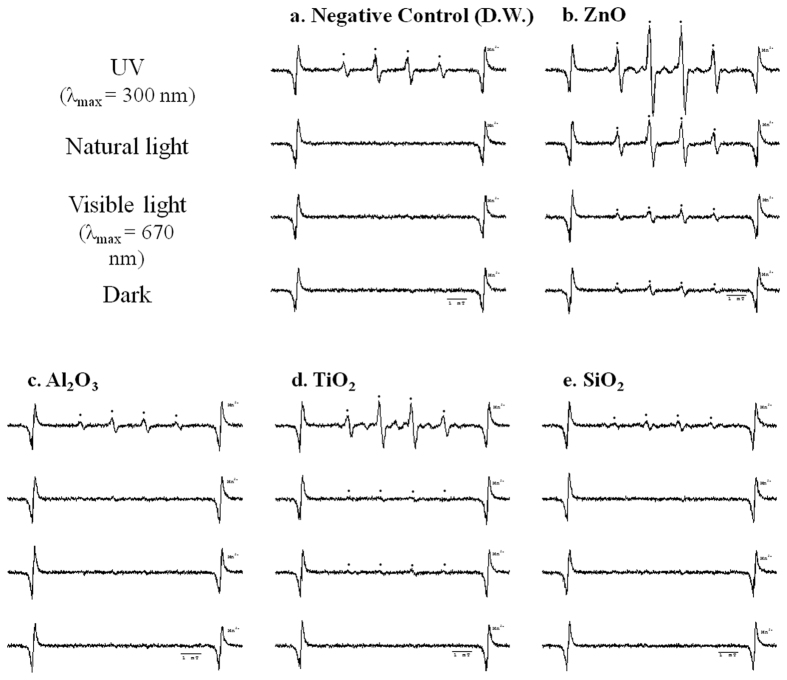
ESR spectra confirming the effects of light exposure interference on sample preparation with DMPO (UV, natural light, visible light, and dark conditions from above). (**a**) ESR spectrum of negative control (DW), (**b**) ESR spectrum of ZnO, (**c**) ESR spectrum of Al_2_O_3_, (**d**) ESR spectrum of TiO_2_, (**e**) ESR spectrum of SiO_2_.

Each nanomaterial was exposed to various light sources, such as UV, natural light, visible light, and dark conditions in the presence of a spin trapping agent (DMPO). The signal of the associated hydroxyl radical adduct was then recorded to determine if any changes occurred. As shown in Fig. 4, all ESR spectra increase in intensity under UV (280–360 nm) conditions as compared to dark conditions. The ESR spectrum of distilled water (DW) without nanomaterials increases under UV conditions as well. Excited H_2_O* generates hydrogen atoms (H^•^) and hydroxyl radicals (^•^OH)^16^, and these hydroxyl radicals are subsequently trapped by DMPO. For this reason, the ESR spectrum of the DMPO-OH adducts increases in intensity.

In the case of TiO_2_, the ESR spectrum increases slightly in visible light (2.03 × 10^13^ spins) and in natural light (2.00 × 10^13^ spins). For ZnO, the ESR spectrum increases in order of dark conditions (2.82 × 10^13^ spins) < visible light (3.37 × 10^13^ spins) < natural light (9.43 × 10^13^ spins) < UV (1.84 × 10^14^ spins), and is more strongly sensitized to light compared to other nanomaterials. The ESR spectra of ZnO and TiO_2_ with photocatalytic behavior increase remarkably under UV conditions. Based on our results, exposure to specific light sources can affect ESR spectra not only for nanomaterials, but for DW as well.

### Effects of ultrasound sonication during sample preparation on ESR spectra

**Figure 5:**
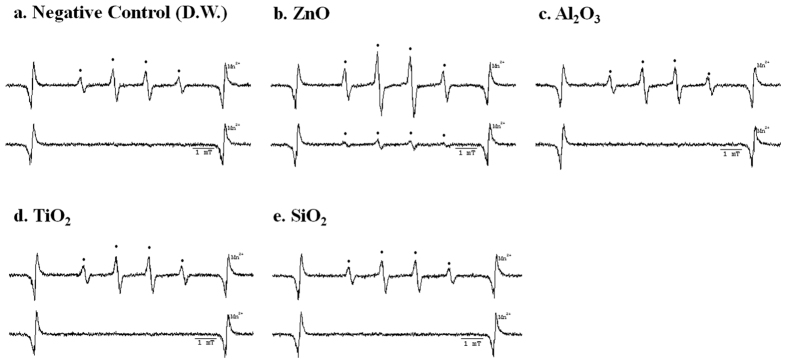
ESR spectra confirming interference effects of ultrasonic dispersion on sample preparation with DMPO (upper: with sonication; bottom: without sonication). (**a**) ESR spectrum of negative control (DW), (**b**) ESR spectrum of ZnO, (**c**) ESR spectrum of Al_2_O_3_, (**d**) ESR spectrum of TiO_2_, (**e**) ESR spectrum of SiO_2_.

Sonication is commonly used in nanotechnology to evenly disperse nanomaterials in liquid media. As shown in Fig. 5, ESR spectra were newly generated after ultrasonic dispersion for D.W., solutions of Al_2_O_3_, TiO_2_, and SiO_2_. In the case of ZnO, the ESR spectrum increases 4.8× when prepared by ultrasonic dispersion compared to a solution without sonication (1.36 × 10^14^ spins vs. 2.82 × 10^13^ spins). Therefore, because ROS signals can be generated by ultrasonic dispersion, such dispersion should not be used for detecting ROS generated from the nanomaterial itself.

## Discussion

ROS are a primary source of nanomaterial toxicity that can form after the exposure of nanomaterials to various elements, and are inherent to the surface conditions of the nanomaterials. However, the *in situ* identification and quantification of ROS in nanomaterials has some obstacles, such as their interactions with repair systems and other unknown factors. To avoid these problems, ROS must be analyzed directly during their formation from nanomaterials. In addition, the direct identification and quantification of ROS by ESR is important in understanding the toxic mechanisms of nanomaterials and in product design integrating these nanomaterials. In particular, the various factors that can affect ESR signals must be considered, such as the characteristics of the parent nanomaterial, spin trapping agent incubation time, and sample preparation. In this report, we examine the magnetic properties, photo catalytic activity, and dispersion method of such nanomaterials and how they can impact ESR analysis, as well as examining samples with and without the parent nanomaterials (removed by centrifugation or filtration) (Fig. 3).

Each spin trapping agents should be applied with a proper incubation time when using ESR to quantify ROS from nanomaterials. In this case, the life span of each spin trapping agent should be considered. Based on our results, the ESR signal intensity of the BMPO adduct decreases after the peak value, in contrast with the DMPO and TPC adducts (Fig. 1). In our results, spin trapping agents have different signal intensities depending on their incubation time (Fig. 1), emphasizing the importance of this parameter.

**Figure 6:**
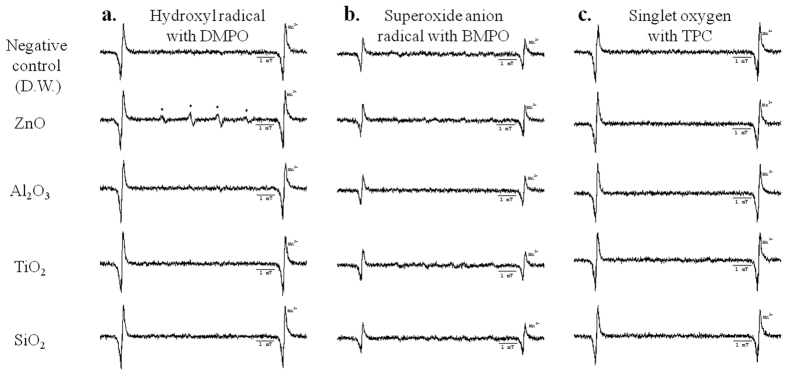
ESR spectra in accordance with well-documented methods in the sample preparation of nanomaterials (negative control, ZnO, Al_2_O_3_, TiO_2_, and SiO_2_ from above). (**a**) ESR data of hydroxyl radical (•OH) production in the presence of DMPO with each nanomaterial, (**b**) ESR data on superoxide anion radical (O_2_^−^) production in the presence of BMPO with each nanomaterial, (**c**) ESR data on singlet oxygen (^1^O_2_) production in the presence of TPC with each nanomaterial.

In the ESR analysis of various metal oxide nanomaterials, well-dispersed nanomaterials such as CoFe_2_O_4_ should not be used for ESR directly due to their inherent magnetic properties, which can affect the ESR data. However, removing the nanomaterials after the reaction of the spin trapping agent is very effective in these cases (Fig. 3a). The photocatalytic activity of the nanomaterials should be additionally considered for ESR. In our results and various reports, the ESR signal intensity of nanomaterials with photocatalytic activity differs before and after light exposure (Fig. 4)^10^,^14^. Based on this result, exposure to various light sources should be also controlled when collecting nanomaterial ESR signals. During sample preparation, ultrasonication can also enhance ESR signals (Fig. 5). Therefore, ultrasonic processes should be carefully applied to ESR, especially in quantitative studies. In our well-documented experimental conditions, only the hydroxyl radical was observed from ZnO nanomaterials. In other cases, there was no observed ROS from nanomaterials (Fig. 6).

## Conclusions

**Figure 7:**
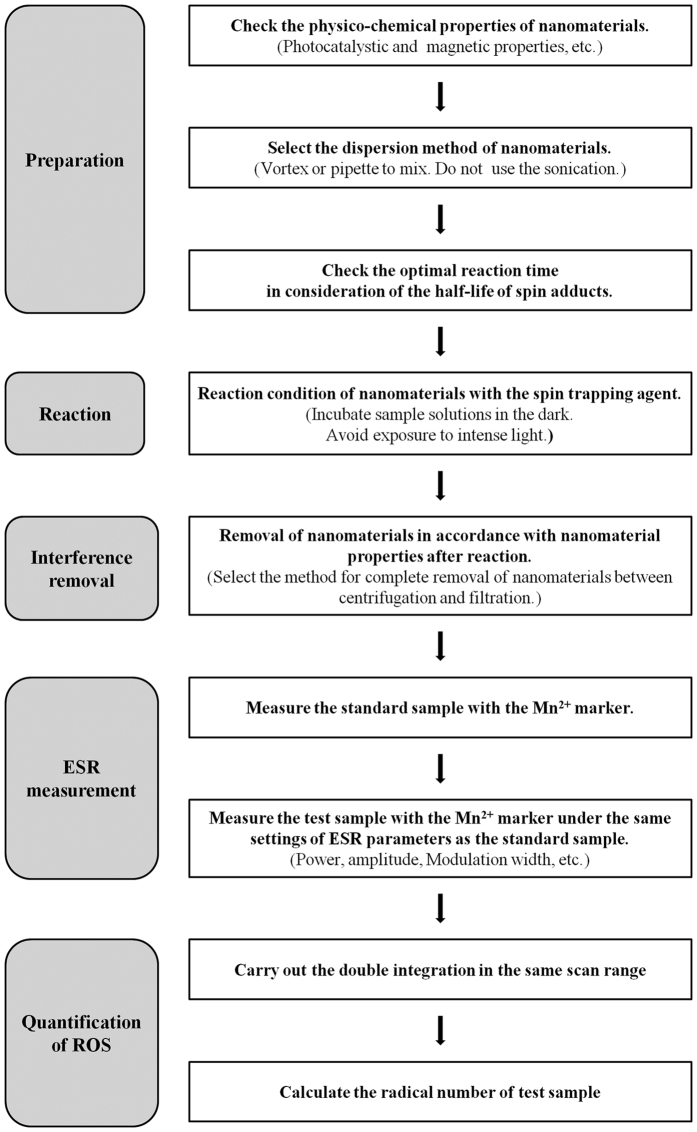
Methodological considerations for using ESR spin-trapping techniques with nanomaterials.

The qualitative and quantitative analyses of ROS generated on nanomaterial surfaces are important for understanding and predicting their toxicity and toxic mechanisms. To this end, ESR spin trapping techniques are very useful tools for detecting ROS formation. However, applying these without considering the physicochemical properties of the nanomaterials and the sampling conditions can lead to data misinterpretation. This study enables researchers to design accurate applications of ESR spin trapping techniques with nanomaterials and to interpret the results of toxicity studies (Fig. 7).

## Materials and Methods

### Materials and instrumental preparation

TiO_2_ and Al_2_O_3_ were purchased from Evonik Industries (Hanau, Germany). Well-dispersed CoFe_2_O_4_, ZnO and SiO_2_ were synthesized at Biterials Co., Ltd. The spin trapping agents were 5,5-dimethyl-1-pyrroline *N*-oxide (DMPO) for •OH, 5-tert-butoxycarbonyl-5-methyl-1-pyrroline-*N*-oxide (BMPO) O_2_^−^, and 2,2,5,5-tetramethyl-3-pyrroline-3-carboxamide (TPC) for ^1^O_2_.

The nitrone spin-trap reagents 5,5-dimethyl-1-pyrroline *N*-oxide (DMPO, 99.9%) and 5-tert-butoxycarbonyl-5-methyl-1-pyrroline-*N*-oxide (BMPO) were purchased from Dojindo (Tokyo, Japan). Sedimented CoFe_2_O_4_, 2,2,5,5-tetramethyl-3-pyrroline-3-carboxamide (TPC), iron(II) sulfate (FeSO_4_), hydrogen peroxide (H_2_O_2_), hypoxanthine (HPX), xanthine oxidase (XOD), diethylenetriaminepentaacetic acid (DTPA), phosphate buffer solution, dimethyl sulfoxide (DMSO) and Rose Bengal were purchased from Sigma-Aldrich (St. Louis, Missouri, USA). 4-hydroxyl-2,2,6,6-tetramethylpiperidine-1-oxyl (TEMPOL) was purchased from JEOL (Tokyo, Japan). Syringe filters (0.20 μm pore size) were purchased from Sartorius (Goettingen, Germany). An ultraviolet (UV)-B lamp (1.6 W, 0.170 A, G8T5E) was purchased from Sankyo Denki (Tokyo, Japan). The visible-light lamp (6 W, 60 mA, SSL-012458) and green lamp (6 W, 60 mA, ST-5030) were purchased from STECH LED (Gyeonggi-do, Korea).

### ESR measurements

The ESR signals of samples were acquired using a JES-TE200 ESR spectrometer (JEOL, Tokyo, Japan) with an X-band standard frequency of 8.8–9.6 GHz. To identify the peaks, the signal components were analyzed by the ES-IPRITS data system with version 3.00 analysis software installed in the ESR instrument. The following ESR parameters were used: a frequency of 9.42 GHz, center field of 335 ± 10 mT, modulation frequency of 100 kHz, time constant of 0.03 s, and power of 5.00 mW. Aqueous samples were loaded into a LC-12 aqueous quartz flat cell (JEOL)^17^.

### Positive control reactions to evaluate the spin trapping agent in each ROS type

#### *Hydroxyl radicals* (•*OH*)

Hydroxyl radicals are generated from the Fenton reaction, which is the oxidation of substrates by iron(II) and hydrogen peroxide. Through this reaction, hydrogen peroxide can produce reactive hydroxyl radicals as Iron(II) is oxidized by hydrogen peroxide to produce iron(III), a hydroxyl radical and hydroxyl ion (Fe^2+^ + H_2_O_2_ → Fe^3+^ + HO + HO)^18^. This reaction mixture contained DW, 0.01 mM FeSO_4_, 50 mM DMPO, and 0.1 mM H_2_O_2_, and the reaction began with the addition of H_2_O_2_. ESR signals were collected every 10 min for 4 h.

#### *Superoxide anion radicals* (*O*_2_^−^)

Superoxide anion radicals were generated from a hypoxanthine–xanthine oxidase (HX-XO) system. The hypoxanthine–xanthine oxidase system generates superoxide anions as shown in reactions 1 and 2^19^.


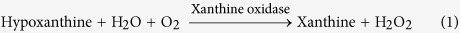



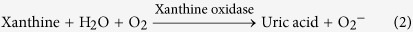


This reaction mixture contained 100 mM phosphate buffer (pH 7.4) with 25 μM DTPA, 0.125 mM hypoxanthine, 50 mM BMPO and 0.125 units/mL of xanthine oxidase. The reaction began with the addition of xanthine oxidase, and ESR signals were collected every 10 min for 4 h.

#### *Singlet oxygen* (^1^ *O*_2_)

The singlet oxygen was generated from the Rose Bengal photosensitizer. When photoexcited, this photosensitizer transfers its energy to the oxygen-producing singlet oxygen (reactions 3 and 4)^20^.


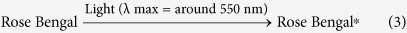



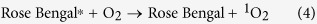


The reaction mixture contained 10 mM TPC and 0.1 mM Rose Bengal in DW; it was illuminated with continuous light (λ max = approximately 550 nm) using an LED lamp for 10 min. ESR signals were collected every 3 min for 1 h.

### Size and morphology measurements of nanomaterials

The morphology and nanostructure of CoFe_2_O_4_ (well-dispersed and sedimented), ZnO, Al_2_O_3_, TiO_2_, and SiO_2_ were characterized by energy-filtering transmission electron microscopy (EF-TEM) using a LIBLA 120 microscope (Carl Zeiss, Oberkochem, Germany) at an accelerating voltage of 120 kV. The suspension (4 mg/mL) of nanomaterials was prepared in methanol and sonicated for 30 s. A portion of the nanomaterial suspension was deposited on a 300-mesh carbon-coated copper grid and dried at room temperature overnight before examination.

### ESR measurements of magnetic nanomaterials

To estimate the effect of magnetic properties on the nanomaterials, we prepared two different states of CoFe_2_O_4_, those being well-dispersed and sedimented materials. Samples were examined by ESR after mixing with a spin trapping agent (DMPO) for 3 h, at which point the hydroxyl radical (•OH) signal was not detected (data not shown). For this reason, samples were detected using a positive control system. After ESR analysis of both samples, we removed the CoFe_2_O_4_ by centrifugation (12,700 × g for 10 min), and then re-analyzed with ESR. Then, to evaluate the interference effects of non-magnetic nanomaterials (ZnO and SiO_2_), ESR spectra were analyzed before and after removing the nanomaterials by centrifugation or filtration. As mentioned above, SiO_2_ was examined with a positive control system, while ZnO nanomaterials were detected without this positive control system.

### ESR measurement to identify light source interference

Each nanomaterial suspension with a spin trapping agent (DMPO) was exposed to UVB (λ_max_ = 280–360 nm), natural light, visible light (λ_max_ = 670 nm), and dark conditions for 3 h, respectively. The signal of hydroxyl radical adduct was recorded to observe and changes compared to dark conditions.

### ESR measurements to identify interference by using ultrasonic dispersion

Mixtures with nanomaterials and spin trapping agent were immersed in an ultrasonic bath (42 kHz, 135 W) for 10 min after reaction for 3 h. All steps were performed in dark conditions. For each mixture, the signal of the hydroxyl radical adduct was recorded to verify if the ultrasonic bath had any effects compared to the unapplied mixture.

### Quantification of ROS

The radical concentrations in the samples were determined by comparing the area of the absorption peak with that of a standard sample (TEMPOL), with 3.0 × 10^14^ spins in 1.0 × 10^−3^ mM TEMPOL as a reference point. Spin counts were calculated from the area of the absorption peak of the Mn^2+^ marker and the signal by double integration of the ESR spectrum. The radical number of the test sample could be calculated relative the area of the standard sample^21^,^22^,^23^. In summary, the standard sample (St) and test sample (T) should be measured with the same ESR spectrometer parameters, respectively. I Double integrating the spectra over the same scan range allows for the quantification of the test sample as


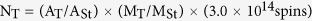


A_St_: The area of absorption peak with a standard sample (1.0 × 10^−3^ mM TEMPOL)

A_T_: The area of absorption peak with a test sample

M_St_: The area of absorption peak of Mn^2+^ marker with a standard sample

M_T_: The area of absorption peak of Mn^2+^ marker with a test sample

N_T_: The radical number of test sample.

## Additional Information

**How to cite this article**: Jeong, M. S. *et al*. Methodological considerations of electron spin resonance spin trapping techniques for measuring reactive oxygen species generated from metal oxide nanomaterials. *Sci. Rep.* **6**, 26347; doi: 10.1038/srep26347 (2016).
